# Transcriptomic and physiological approaches to decipher cold stress mitigation exerted by brown-seaweed extract application in tomato

**DOI:** 10.3389/fpls.2023.1232421

**Published:** 2023-09-11

**Authors:** Matteo Borella, Ali Baghdadi, Giovanni Bertoldo, Maria Cristina Della Lucia, Claudia Chiodi, Silvia Celletti, Saptarathi Deb, Andrea Baglieri, Walter Zegada-Lizarazu, Elena Pagani, Andrea Monti, Francesca Mangione, Francesco Magro, Christian Hermans, Piergiorgio Stevanato, Serenella Nardi

**Affiliations:** ^1^ Department of Agronomy, Food, Natural Resources, Animals and Environment (DAFNAE), University of Padua, Padua, Italy; ^2^ Department of Agricultural and Food Sciences (DISTAL), University of Bologna, Bologna, Italy; ^3^ Department of Life Sciences (DSV), University of Siena, Siena, Italy; ^4^ Department of Agriculture Food Environment (Di3A), University of Catania, Catania, Italy; ^5^ Sipcam Italia S.p.A. Belonging Together with Sofbey SA to the Sipcam Oxon S.p.A. Group, Pero, Italy; ^6^ Crop Production and Biostimulation Laboratory (CPBL), Brussels Bioengineering School, Universitè libre de Bruxelles, Brussels, Belgium

**Keywords:** biostimulant, brown seaweed extract, cold stress, transcriptome, plant physiology, antioxidant molecules, tomato

## Abstract

Chilling temperatures represent a challenge for crop species originating from warm geographical areas. In this situation, biostimulants serve as an eco-friendly resource to mitigate cold stress in crops. Tomato (*Solanum lycopersicum* L.) is an economically important vegetable crop, but quite sensitive to cold stress, which it encounters in both open field and greenhouse settings. In this study, the biostimulant effect of a brown-seaweed extract (BSE) has been evaluated in tomato exposed to low temperature. To assess the product effects, physiological and molecular characterizations were conducted. Under cold stress conditions, stomatal conductance, net photosynthesis, and yield were significantly (p ≤ 0.05) higher in BSE-treated plants compared to the untreated ones. A global transcriptomic survey after BSE application revealed the impact of the BSE treatment on genes leading to key responses to cold stress. This was highlighted by the significantly enriched GO categories relative to proline (GO:0006560), flavonoids (GO:0009812, GO:0009813), and chlorophyll (GO:0015994). Molecular data were integrated by biochemical analysis showing that the BSE treatment causes greater proline, polyphenols, flavonoids, tannins, and carotenoids contents.The study highlighted the role of antioxidant molecules to enhance tomato tolerance to low temperature mediated by BSE-based biostimulant.

## 1 Introduction

Low-temperature stress is a common challenge faced by warm-climate plants that can dramatically impact the yield. Although temperatures are generally increasing, late and out-season frosts are also happening often, and affect the potential distribution of warm-climate-adapted plants. Warm-climate plants can be harmed by chilling temperatures (0-12°C) and critically damaged by freezing temperatures (< 0°C). When the temperature drops below 10°C, plant growth and development are inhibited, and photosynthesis is compromised (Shi et al., 2016; Ding et al., 2017), while temperatures below 0°C can cause ice formation in the intercellular spaces of plant tissues provoking cell membrane disruption (Kidokoro et al., 2022).

Plants have evolved different strategies to cope with cold stress. The first cold-induced reaction is the accumulation of reactive oxygen species (ROS) *i.e.*, OH·, O_2_·, H_2_O_2_, which act as signal molecules in response to several stresses but also are toxic by-products that need to be worked off. To alleviate oxidative stress under cold conditions, detoxification mechanisms have been implemented such as the production of antioxidant enzymes, and a major activity of AOX (alternative oxidase) over COX (cytochrome c oxidase) (Heidarvand and Amiri, 2010; Ding et al., 2017). Low temperatures are also increasing the production of flavonoids (Jaakola and Hohtola, 2010). These pigments are divided into anthocyanins, flavones, flavonols, and isoflavonoids. They stimulate DNA repair and protect against oxidative stress (Hichri et al, 2011; He et al., 2022). Among the molecular determinants involved in cold acclimation, the *C-REPEAT BINDING FACTOR* (*CBF*) transcription factor and the *INDUCER OF CBF EXPRESSION* (*ICE*) form the *ICE-CBF* signaling pathway, which plays a pivotal role in plant acclimation to cold controlling the expression of *COLD REGULATED* (*COR*) genes (Liu et al., 2012; Shi et al., 2016; Hwarari et al., 2022; Kidokoro et al., 2022; Gusain et al., 2023).

Tomato (*Solanum lycopersicum* L.) is the second most important vegetable crop in the world, next to potato. Cultivated over 5.16 10^6^ ha, it produces 189 10^6^ t of fresh fruit, with an annual value exceeding 90 10^9^ USD (FAOSTAT, 2023). Native to Western South America (Rodríguez et al., 2011), tomato is grown all over the year, worldwide, in open field or in greenhouses. Particularly, in the Mediterranean regions tomato is generally cultivated under unheated greenhouses to obtain year-round production. Two short cycles are often completed per year (autumn and spring). In these conditions, tomato plants can frequently experience cold stress (Brazel and Graciet, 2023). Nonetheless, climate change is threatening tomato production and thus, improving tolerance towards extreme temperatures is a matter of concern.

Tomato growth is optimal with an average daily temperature ranging between 18 and 25°C, and a night temperature between 10 and 20°C. Temperatures above 32°C and below 12°C induce growth retardation and impact fruit quality (Mesa et al., 2022). Due to its geographical origin, tomato is chilling sensitive and it shows poor ability to acclimate to cold (Barrero-Gil et al., 2016).

Some of the cold response mechanisms identified in other plants have been reported in tomato as well, even though in this crop, the pathways leading to low-temperature adaptation are still mostly unknown. Liu et al. (2020) identified SlGRAS4, a transcription factor promoting cold tolerance in tomato. The Sl*GRAS4* pathway seems to work in parallel and independently from the *ICE1* one. Sl*GRAS4* has also been shown to increase the expression of antioxidant-coding genes, underlining again a very similar effect as Sl*ICE1* (Liu et al., 2020).

The potential of biostimulants in agriculture has widely been reported: these products can stimulate plant growth, sustain yield, improve crop quality, and contribute to tolerance towards environmental stressors and pathogens (Van Oosten et al., 2017; Meddich, 2022). The seaweed extracts (SWE) are forming an important class of biostimulants. Precisely, they can promote cold tolerance and prevent cold-stress damage (Van Oosten et al., 2017). Many studies claim that SWE can increase cold tolerance (Digruber et al., 2018; Ali et al., 2021; Lakshmi and Meenakshi, 2022), but very few are actually reporting a scientific study. Some examples are the use of *Ascophyllum nodosum* in barley (Burchett et al., 1998), tobacco (Zamani-Babgohari et al., 2019), and Arabidopsis (Rayirath et al., 2009; Nair et al., 2012). Thus, the mode of action of SWE in the mitigation of cold stress remains not clear, even though these products seem to enhance membrane integrity and protect chlorophyll. The micronutrients contained in SWE might also protect against oxidative stress (Van Oosten et al., 2017). Particularly, *A. nodosum* seems to help to maintain membrane integrity thus reducing electrolyte leaking, and modulating the expression of cold-responsive genes (*COR15A*, *RD29A*, and *CBF3*) (Rayirath et al., 2009; Shukla et al., 2019). A lot is at stake about biostimulants and cold-stress responses, but very little consistent information is available.

To fill this gap, the transcriptomic and physiological responses to a commercially available brown seaweed extract (BSE)-based biostimulant were investigated on tomato plants exposed to cold stress. The effect of BSE foliar application was tested on photosynthetic activity and yield components. Furthermore, molecular targets of BSE were identified by RNA-Seq analysis. Finally, the products of most representative genes were quantified. Our findings provide valuable insights for the development of sustainable and effective strategies to enhance tomato production under cold stress conditions, in open field, and in greenhouse.

## 2 Materials and methods

### 2.1 Plant material and growing conditions

Tomato (*Solanum lycopersicum* L., cv. Micro-Tom) seeds were germinated in a tray filled with a commercial substrate (TS4 Klasmann-Deilmann, Germany) consisting of 35% (w:w) white sod peat, 45% white peat, 15% perlite and 5% peat fiber. Seedlings were fertigated once a week with IDROFEED 20-20-20 NPK (Tiller, Italy) at a dose of 1 g L^-1^. Plants with two to four leaves were transfered in pots with 1.2 L capacity and supplied with the same growth medium. The photoperiod was 15 h–19 h, with light intensity (PFD) of 400 µmol photons m^-2^ s^-1^, temperatures were ranging from 24°C during the day to 20°C at night and relative humidity was kept constant to 60%. Pots were supplied with 150 mL of ultra-pure water twice a week.

Plants were treated three times, at phenological stages corresponding to BBCH51 (first inflorescence visible), BBCH61 (first flower open) and BBCH65 (first flower of the fifth inflorescence open). At each of the three stages, half of the plants were treated through foliar spray with a solution containing 2.75 g L^-1^ (recommended dosage from the producer) of a brown seaweed extract (BSE) provided by Sipcam-Oxon (Pero, Italy). The remaining plants were treated with an equal volume of ultra-pure water as an untreated control. Two days after the last BSE foliar application, a sub-set of treated and untreated plants were exposed to 4°C during three successive nights in a cold chamber. During the day plants were moved back to the growth chamber. The same experiment was repeated three times for transcriptomics and biochemical analysis. The experiments were carried out during 2021.

### 2.2 Leaf gas exchange and yield components

Stomatal conductance and net photosynthesis were measured on the youngest fully expanded leaf of six plants per experimental condition, using an infrared gas analyzer (CIRAS 3 PP Systems, Amesbury, MA, USA) as described in Baghdadi et al. (2022). Measurements were done before the cold exposure and after 48 h, 72 h, and 96 h.

At ripening, tomato fruits were harvested. The number and diameter of fruits, as well as their fresh and dry weight were determined for each plant. The fruits horizontal diameter was manually measured with a caliper at the highest diameter along the fruit equator. Dry weights were recorded after oven-drying the samples at 105°C.

### 2.3 RNA extraction and library preparation

Young mature leaves of six plants per experimental condition were harvested 24 h (T1) and 48 h (T2) after cold stress exposure. 50 mg of leaf tissue were collected and immediately frozen in liquid nitrogen and stored at -80°C. Total RNA was extracted using a Maxwell 16 LEV Plant RNA Kit (Promega Corporation, USA) from leaf tissues, ground in liquid nitrogen with a tissue homogenizer. Next, mRNA was isolated from 1 µg of total RNA, using the Dynabeads mRNA DIRECT Micro Kit (Thermo Fisher Scientific, Carlsbad, CA, United States). Transcriptome RNA libraries were prepared with the Ion Total RNA-Seq Kit v2 (Thermo Fisher Scientific) following the manufacturer’s instructions. The yield and size distribution of cDNA barcoded libraries were checked using D1000 screen Tape (Agilent Technologies, USA) and they were normalized, pooled, and sequenced on an Ion Torrent S5 with an Ion 540 chip kit (Thermo Fisher Scientific). Single-end sequencing (200 bp) was performed to achieve an average of 8 10^6^ reads per sample.

### 2.4 Transcriptomic data analysis

Raw single end reads were quality checked using FastQC v0.11.9 (https://www.bioinformatics.babraham.ac.uk/projects/fastqc/) and mapped to the *Solanum lycopersicum* L. reference genome SL3.0 (NCBI assembly: GCA_000188115.3) using bowtie2 v2.3.5.1 (Langmead and Salzberg, 2012). Aligned files were processed using samtools suite (Li et al., 2009) to calculate the read counts for each gene. Raw data were normalized based on library size and underwent a Variance Stabilization Transformation (VST) using DESeq2 (Love et al., 2014) R-package. The VST matrix was used to calculate the Euclidean distances between samples and to investigate the grouping of samples in reduced principal components space at each sampling time, with potential outliers identified and excluded from further analysis.

The differential expression analysis (DEA) tested each sampling time group of samples separately (**Supplementary Figure 1**), to avoid batch effects. It was performed to compare gene expression profiles between samples under two experimental conditions. Two T1 and T2 sampling times, were evaluated separately to determine differential expression between treated and control samples. The DESeq2 (Love et al., 2014) R-package was used to perform DEA. A Generalized Linear Model (GLM) with a Gamma-Poisson distribution was fitted to the data, and Wald’s test was used to determine statistical significance. Differentially expressed genes (DEGs) were identified as those with a raw p-value ≤ 0.05 and |log_2_(fold change)| > 1.

ShinyGO web tool v 0.75 (http://bioinformatics.sdstate.edu/go/) (Ge et al., 2020) was used to group DEGs into biological categories. Biological processes (BP) and KEGG (Kanehisa and Goto, 2000) pathway results with a significant threshold (FDR ≤ 0.05) were considered for the analysis. The resulting data were integrated with NCBI’s gene description for further considerations (NCBI, 1988).

### 2.5 Determination of proline content

The proline content was estimated according to the method of Bates et al. (1973) (Quagliata et al., 2023). Briefly, 0.1 g fresh weight (FW) of tomato leaves were homogenized with 2 mL of 3% (w:v) 5-sulfosalicylic acid dihydrate. After a centrifugation step at 5000 rpm for 10 min, an aliquot (0.5 mL) of the supernatant was added to reaction tubes containing an equal volume of glacial acetic acid and acid-ninhydrin reagent (previously prepared by dissolving 1.25 g ninhydrin in 30 mL glacial acetic acid and 20 mL 6 M phosphoric acid). The reaction was conducted at 100°C for 1 h and stopped by cooling the samples in ice. The reaction mixture was extracted with 1.5 mL toluene and shaken vigorously for 20 sec. Subsequently, the chromophore containing toluene was separated from the aqueous phase and the absorbance read at 520 nm with an Agilent UV-Vis 8453 spectrophotometer (Santa Clara, CA, USA), using toluene as a blank. Calibration was done with 2 – 600 µL of a 1 mM L-proline (98.5 - 101.0%, pharma grade, PanReac AppliChem ITW Reagents S.R.L., Monza, Italy) stock solution, and the results were expressed as µmol g^-1^ FW. Measurements were taken from 31 different plants.

### 2.6 Determination of total phenolic, total flavonoid compounds, and condensed tannins content

The contents of total phenolics (TPC), flavonoids (TFC), and condensed tannins were determined in the extracts of tomato leaves, previously dried in the dark, according to Wakeel et al. (2019) with some modifications. A total of 34 plants were considered for these measurements. For the extraction, 1 g DW of leaf material was soaked in 10 mL of 80% (v:v) methanol. The samples were placed on an orbital shaker (ASAL VDRL mod. 711, Cernusco s/N, Milano, Italy) for 30 min and then incubated in the dark at 4°C. After 48 h of incubation, the samples were filtered through Whatman filter paper no. 1 and the filtrates were used for TPC, TFC, and condensed tannin assays.

The TPC was quantified using the Folin-Ciocalteu method (Al-Duais et al., 2009). Briefly, 0.125 mL of leaf extract was added to 2 mL of water, followed by the addition and mixing of 0.125 mL of the Folin-Ciocalteu’s reagent. The samples were left for 3 min in the dark and then 1.250 mL of 7% (w:v) Na_2_CO_3_ and 1 mL of distilled H_2_O were added and shaken vigorously followed by 90 min incubation in the dark. Then, the absorbance of the blue solutions was read at 760 nm with an Agilent UV-Vis 8453 spectrophotometer (Santa Clara, CA, USA). The amount of the extract was substituted by the same amount of 80% (v:v) methanol in the blank. Gallic acid (98%, Thermo Fisher Scientific Inc., Rodano, Milano, Italy) (in the 5 – 300 µg mL^-1^ concentration range) was the standard of choice and the results were expressed as gallic acid equivalent (GAE) mg g^-1^ DW of extract.

The TFC was quantified with an aluminum chloride colorimetric method (Chang et al., 2002). Briefly, 0.250 mL of leaf extract were mixed with 0.075 mL of 5% (w:v) NaNO_2_ and 5 min later with 0.075 mL of 10% (w:v) AlCl_3_. The samples were shaken and after 5 min of incubation in the dark were neutralized with 0.5 mL of 1 M NaOH solution. The mixtures were left in the dark for 15 min and then the readings were taken at 415 nm with an Agilent UV-Vis 8453 spectrophotometer (Santa Clara, CA, USA) against a blank of 80% (v:v) methanol. Quercetin (≥ 95%, Merck KGaA, Darmstadt, Germany) (in the 12.5 – 150 µg mL^-1^ concentration range) was the standard of choice and the results were expressed as quercetin equivalent (QE) mg g^-1^ DW of extract.

The condensed tannin content was quantified using the acidified vanillin method (Broadhurst and Jones, 1978). Briefly, 0.5 mL of leaf extract were mixed with 3 mL of 4% vanillin in methanol and 1.5 mL of concentrated HCl. The mixtures were incubated in the dark for 20 min and then read at 500 nm with an Agilent UV-Vis 8453 spectrophotometer (Santa Clara, CA, USA) against a blank of 80% (v:v) methanol. Tannic acid (ACS reagent, Merck KGaA, Darmstadt, Germany) (in the 12.5 – 900 µg mL^-1^ concentration range) was the standard of choice and the results were expressed as tannic acid equivalent (TAE) mg g^-1^ DW of extract.

### 2.7 Determination of leaf pigments content

The content of pigments (chlorophyll *a*, chlorophyll *b*, and carotenoids) was measured in leaves of tomato plants sampled 48 h after the chilling exposure, following the method of Prodhan et al. (2017) with slight modifications. Four mL of chilled methanol were added to 0.050 g FW of leaf material. The mixture was homogenized and incubated for 30 min in the dark at 4°C. Afterwards, the samples were centrifuged (PK110 centrifuge, Alc International S.r.l., Cologno Monzese, MI, Italy) at 3500 rpm for 20 min. The absorbance of supernantants were measured at 470, 653 and 666 nm with an Agilent UV-Vis 8453 spectrophotometer (Santa Clara, CA, USA). The specific absorption coefficient in methanol was used to calculate chlorophyll a and b and total carotenoid contents in leaves. The results were expressed as mg g^-1^ FW (Lichtenthaler and Wellburn, 1983).

### 2.8 Statistical analysis

A Wilcoxon rank sum test was employed to compare the physiological parameters between the experimental conditions, with a significance threshold of p ≤ 0.05. Principal Component Analysis (PCA) was conducted on all variables related to the physiological responses. RStudio software (v. R-4.2.3) was used for statistical analysis and for plotting the results.

## 3 Results

### 3.1 The BSE treatment increases photosynthesis and fruit yield during control and cold stress conditions

**Figure 1:**
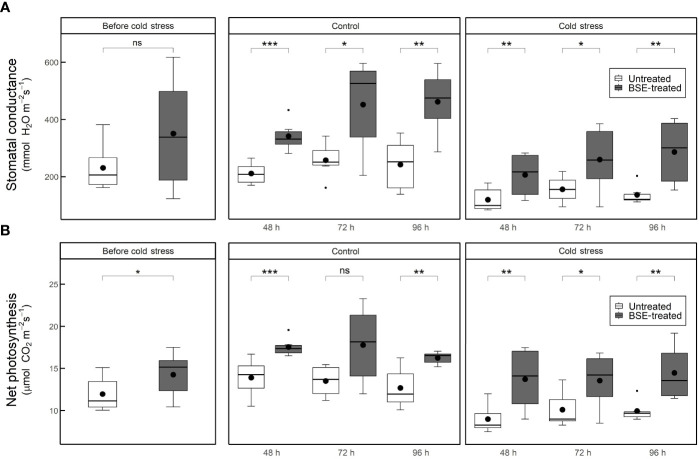
Stomatal conductance **(A)** and net photosynthesis **(B)** in cold-stress and control conditions, in treated and untreated plants. Parameters were measured at 4 different time points: before the cold exposure, and 48, 72 and 96 hours after the cold exposure. Box plots show medians, 25^th^ and 75^th^ percentiles, and non-outlier ranges. Small dots are considered outlier observations, big dots represent the average values. Significance is based on Wilcoxon’s test: ns, not significant, *p-value < 0.1, **p-value < 0.05, ***p-value < 0.01.

Stomatal conductance and net photosynthesis were measured in tomato plants untreated or treated with BSE, in control or cold stress conditions. Measurements were taken prior cold stress application and 48 h, 72 h and 96 h after. The BSE treatment significantly (p < 0.05) increased both parameters (**Figure 1**), and this is consistent with previous report (Baghdadi et al., 2022). At the end of the treatment, the stomatal conductance increased by 69.6% and 73.8% (**Figure 1A**), and the net photosynthesis by 26.1% and 37.0% (**Figure 1B**) between untreated and BSE-treated plants in control and cold stress conditions, respectively.

**Figure 2:**
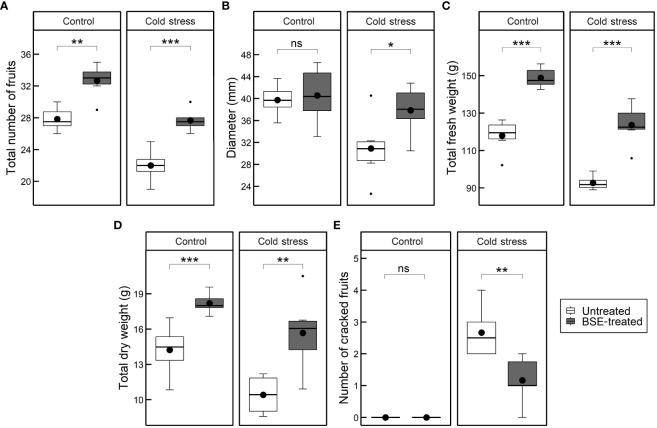
Changes in the yield. Total number of cracked fruits **(A)**, fruits diameter **(B)**, total fresh weights of fruits **(C)**, total dry matter of fruits **(D)**, and number of cracked fruits **(E)** in cold-stress and control conditions, in treated and untreated plants. Box plots show medians, 25^th^ and 75^th^ percentiles, and non-outlier ranges. Small dots are considered outlier observations, big dots represent the average values. Significance is based on Wilcoxon’s test: ns, not significant, *p-value < 0.1, **p-value < 0.05, ***p-value < 0.01.

Yield traits like the number, size, and weight of fruits were measured (**Figure 2**). The BSE treatment significantly (p < 0.05) increased the total number of fruits under both control (+17.4%) and cold (+25.8%) conditions (**Figure 2A**). In cold conditions, the BSE treatment caused a significant increment of 22.55% of the fruit diameter (**Figure 2B**). The BSE treatment resulted in a significant average increase in fresh weight under both control (+26.2%) and cold stress (+33.4%) conditions (**Figure 2C**). The average total fruit dry matter of BSE-treated plants increased under both control (+27.9%) and cold stress (+50.4%) conditions (**Figure 2D**). Finally, the treatment did not affect the number of cracked fruits in control conditions, since no fruits cracked, but it significantly reduced (-56.2%) the number of cracked fruits under cold conditions (**Figure 2E**). Other characteristics analysed were not affected by the treatment (**Supplementary Table 1**).

**Figure 3:**
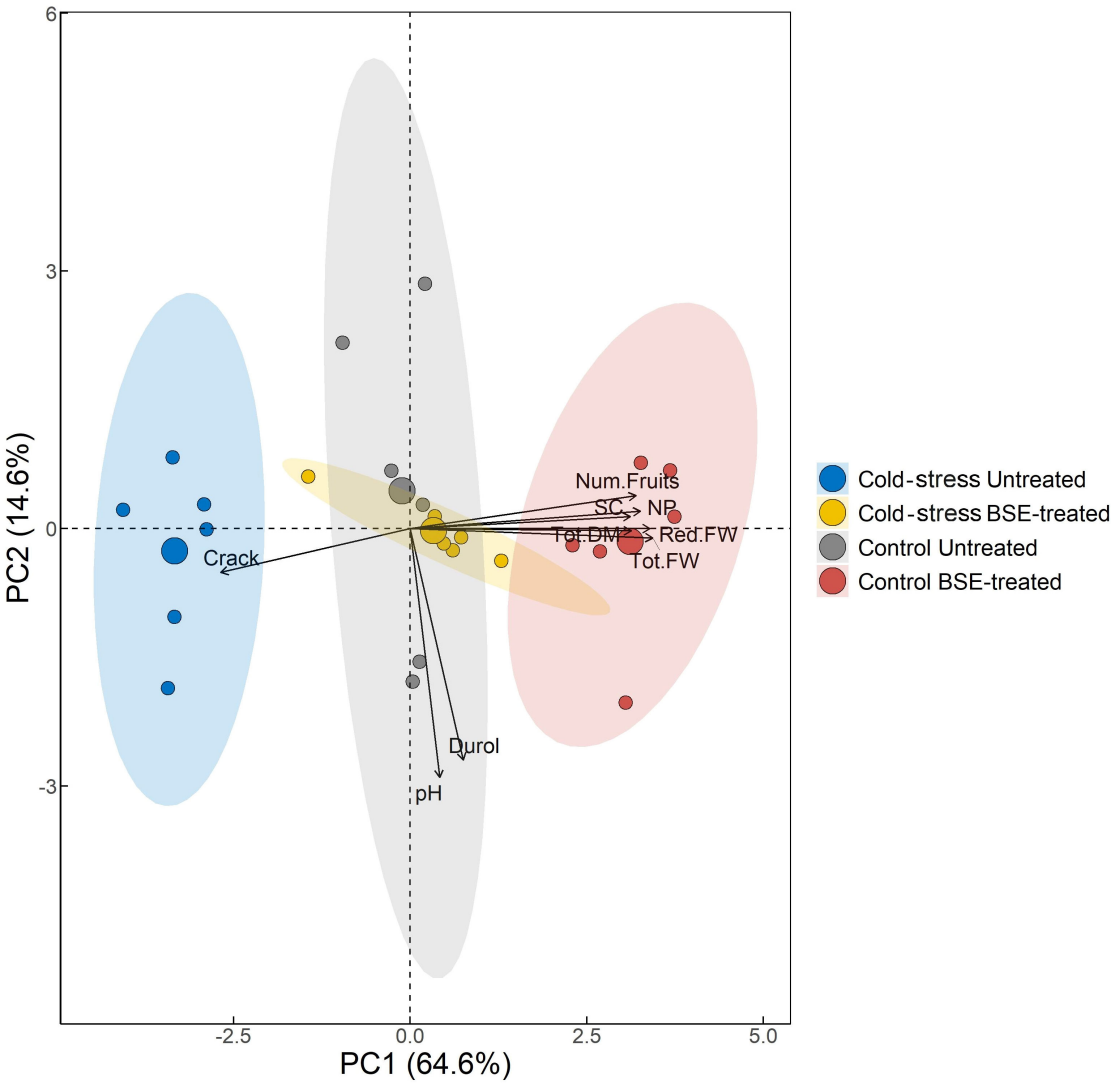
Principal Component Analysis. The variables used to compute PCs and their correlation and contribution with the firsts two PCs are shown. Points are colored by the group. The percentage in each axis shows how much variability each principal component was able to explain. The direction of the arrows and their color refers to the correlation and to the contribution of each variable have with the first two PCs respectively. In the figure: red fruits weight (Red.FW), total fruits weight (Tot.FW), frutis dry weight (Tod.DM), fruits pH (pH), fruits hardness (Durol), number of cracked fruits (Crack), stomatal conductance (SC), net photosynthesis (NP), fruits number (Num.Fruits).

PCA was performed to obtain an overview of the global physiological change between BSE-treated plants and untreated ones, with cold stress imposition and in control conditions (**Figure 3**). PC1 explained 64.6% of the total observed variation and it is the one that describes the two variables of interest, lower values of PC1 are associated with cold-stressed samples and untreated ones, while high values refer to BSE-treated samples under control temperature conditions. It was found that bio-stimulated plants presented a distinct physiological profile with higher values of fresh weight, dry matter, SC, NP, and the number of fruits, and fewer cracked fruits. The table underlying the graph shows that the variables as fresh weight, dry matter, SC, NP, and the number of fruits are positively correlated with PC1, stating that the biostimulant application promotes higher levels of these parameters both in cold and in control temperature conditions. It is possible to follow the same logic for the Crack variable stating that it is less likely to have cracked fruits with biostimulant treatment application (**Supplementary Table 1**).

### 3.2 The BSE treatment affects proline and phenols metabolisms under cold stress

Transcriptomic analysis has been performed 24 (T1) and 48 (T2) hours after the cold stress, in BSE-treated and untreated plants. The total number of mapped reads was 96,434,292 with an average of 8,036,191 reads per sample. The average alignment rate was 62.7% (**Supplementary Table 2**; **Supplementary Figure 2**).

**Figure 4:**
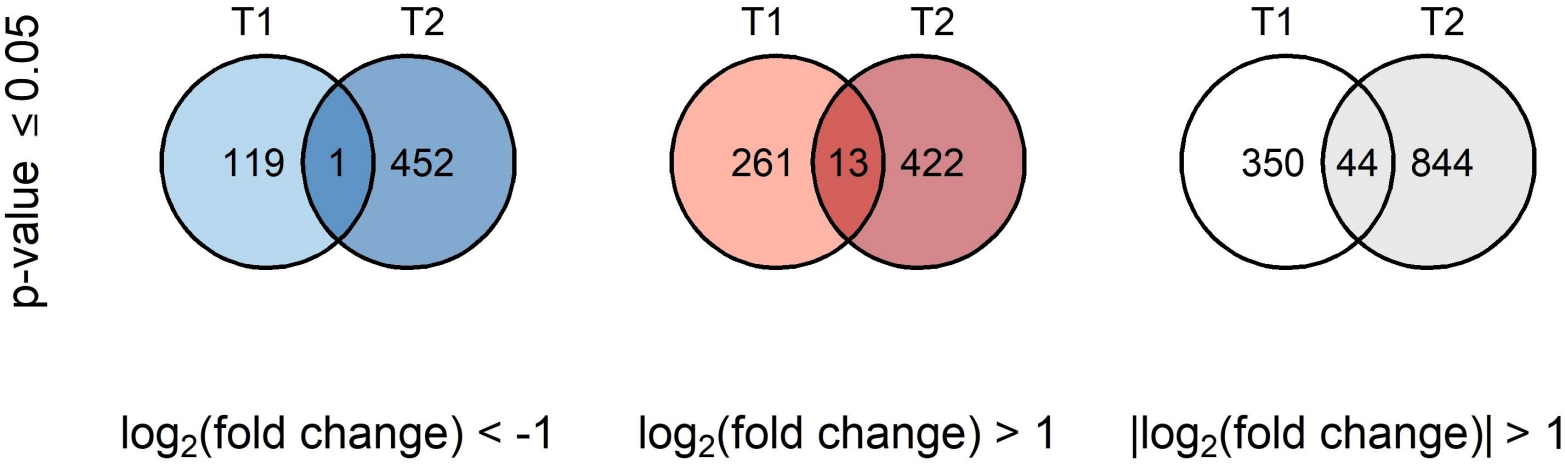
Venn’s diagrams of common DEGs. Blue diagrams show downregulated genes, red ones upregulated genes while the last white diagram shows all the differentially expressed genes.

The BSE treatment greatly affected the transcriptome profile of cold-stressed plants. A total of 394 and 888 genes were differentially expressed after 24 h and 48 h, respectively (**Figure 4**). Only one gene was down-regulated and 13 genes were up-regulated, persistently at both time points. Thirty genes presented opposite expression pattern over time (**Table 1**).

**Table 1:** Genes with inverted tendency in the expression from T1 to T2 (p-value ≤ 0.05).

Gene ID	Symbol	LFC_T1_	LFC_T2_	Gene annotation (Sol Genomics)
Solyc01g106620	PR1	1.614	-2.372	Pathogenesis-related protein 1a
Solyc02g085020	DFR	-1.422	1.112	Dihydroflavonol 4-reductase
Solyc02g085980	–	3.649	-2.651	Unknown Protein
Solyc02g085990	–	1.663	-2.163	Unknown Protein
Solyc02g086000	–	5.389	-2.651	Unknown Protein
Solyc02g086040	–	2.638	-2.651	Monoglyceride lipase
Solyc02g089780	SNL6	-1.267	1.383	Cinnamoyl-CoA reductase-like*
Solyc02g092550	LOB38	1.843	-1.986	Lateral organ boundaries domain protein 38
Solyc02g093070	–	1.348	-2.201	Oxoglutarate and iron-dependent oxygenase
Solyc03g006490	–	1.002	-1.053	Aluminum-induced protein-like
Solyc03g020030	–	1.177	-1.764	Proteinase inhibitor II
Solyc03g115540	bHLH024	1.035	-1.022	BHLH transcription factor
Solyc03g115930	–	1.298	-1.375	Calmodulin-like protein
Solyc03g116690	–	1.151	-1.378	Blue copper protein
Solyc04g007800	–	-1.028	1.032	C2 domain-containing protein
Solyc04g063270	PRR	1.196	-1.244	Pentatricopeptide repeat-containing protein
Solyc04g082500	–	1.084	-1.056	ATP binding/serine-threonine kinase
Solyc05g010320	CHI1	-1.083	1.510	Chalcone–flavonone isomerase 1
Solyc05g052240	CHI3	-1.368	1.729	Chalcone–flavonone isomerase 3
Solyc05g053550	CHS2	-3.332	1.368	Chalcone synthase 2
Solyc06g059710	–	1.072	-1.855	Stearoyl-acyl carrier protein desaturase
Solyc08g066050	–	1.131	-1.741	Serine/threonine-protein kinase 6
Solyc08g080590	OSM81	1.192	-1.391	Osmotin 81
Solyc08g082470	–	1.178	-3.030	Harpin-induced protein
Solyc09g006005	–	1.185	-1.384	Pathogenesis-related leaf protein 4*
Solyc09g059170	–	-1.241	1.438	Anthocyanidin 3-O-glucosyltransferase
Solyc09g091510	CHS1	-2.034	1.060	Chalcone synthase 1
Solyc10g083440	–	-1.632	1.166	UDP flavonoid 3-O-glucosyltransferase
Solyc11g013110	ANS	-1.610	1.418	Anthocyanidin synthase
Solyc12g011010	–	2.546	-2.564	Meiosis 5

log_2_(fold change) (LFC) of each gene is shown. ITAG 3.2 IDs were obtained blasting the FASTA sequence of the mRNA to Sol Genomics website and selected the homologous with a score ≥ 200 and the highest id%.

*unavailable annotation on Sol Genomics, NCBI annotation was used.

**Figure 5:**
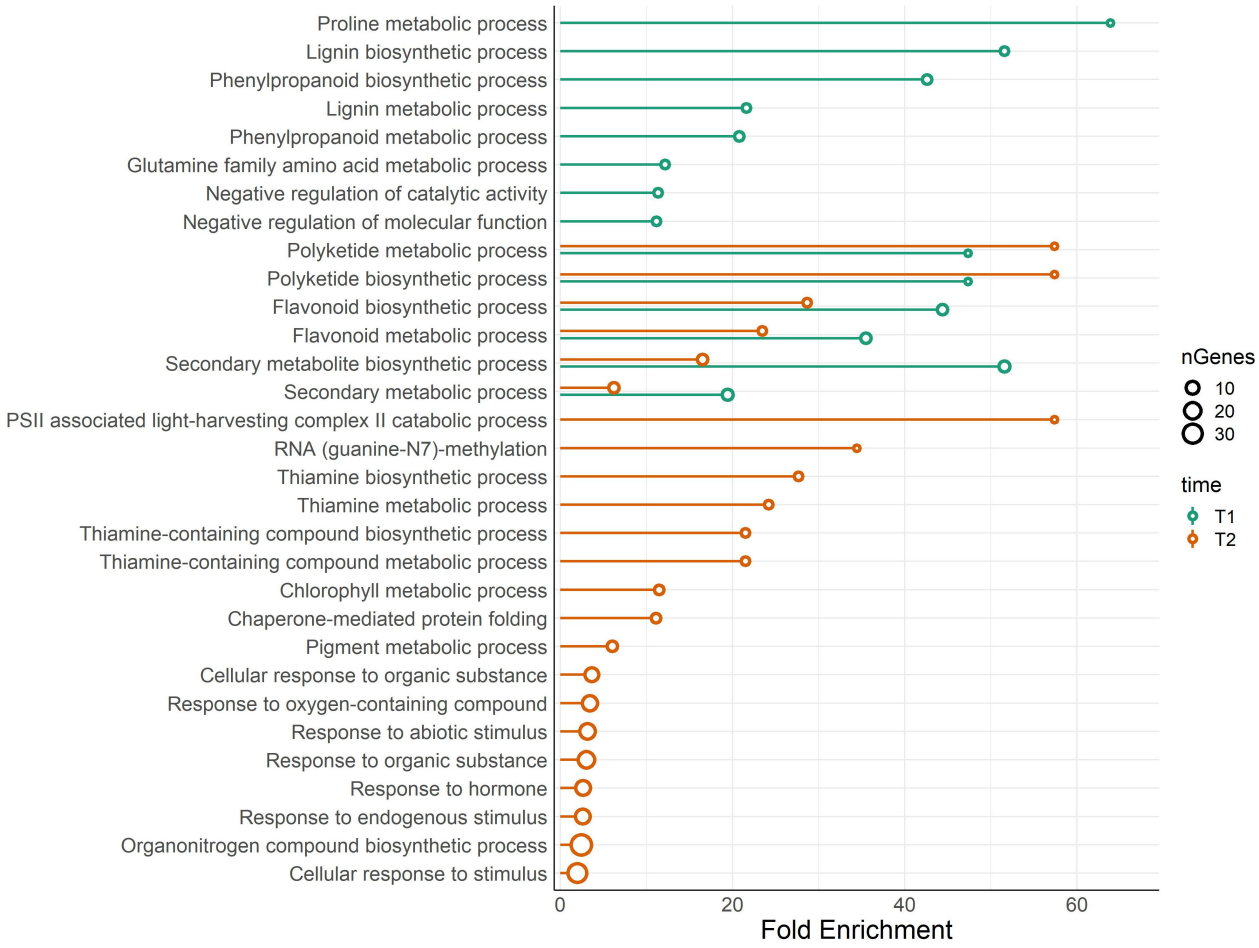
Lollipop plot of biological processes related to DEGs. The analysis compares BSE-treated plants with respect to untreated plants, in cold stress situation. Colors of the bars show the results for the DEGs at T1, and T2, while the size of the dots refers to the number of genes that belong to that ontology. X-axis shows the fold enrichment of each pathway (FDR ≤ 0.05).

One Gene Ontology (GO) analysis was conducted with differentially expressed genes (DEG) between BSE and control treatments under cold stress (**Figure 5**). Significantly enriched GO terms included proline metabolic process (GO:0006560), flavonoid metabolic (GO:0009812) and biosynthetic processes (GO:0009813), polyketide biosynthetic (GO:0030639) and metabolic (GO:0030638) processes. Four pathways related to thiamine (GO:0009228, GO:0006772, GO:0042723, GO:0042724 - thiamine biosynthetic/metabolic process, thiamine-containing compound biosynthetic/metabolic process, respectively), chlorophyll metabolic process (GO:0015994) and pigment metabolic process (GO:0042440). Genes related to these pathways are indicated in **Table 2**.

**Table 2:** DEGs with significantly enriched ontologies.

Gene ID	Symbol	LFC_T1_	LFC_T2_	Gene annotation (NCBI)
Genes involved in the proline metabolism				
Solyc02g089630	PDH1	-1.925	–	Proline dehydrogenase
Solyc06g019170	-	1.310	–	Gamma-glutamyl phosphate reductase
Genes involved in flavonoids metabolism				
Solyc02g083860	F3H	-1.610	–	Flavanone 3-hydroxylase
Solyc03g117600	HCT	1.062	–	Hydroxycinnamoyl-CoA shikimate
Solyc05g053550	CHS2	-3.332	1.367	Chalcone synthase 2
Solyc09g091510	CHS1	-2.034	1.060	Chalcone synthase 1
Solyc11g013110	ANS	-1.609	1.417	Anthocyanidin synthase
Genes involved in the thiamine metabolism				
Solyc06g006080	THIC	–	-1.258	Thiamine biosynthesis protein
Solyc07g064160	Thi4	–	-1.227	Thiazole biosynthetic enzyme
Genes involved in pigments metabolism				
Solyc01g086650	–	–	-1.049	Siroheme synthase
Solyc07g024000	–	–	-1.245	Dehydrogenase/reductase 3
Solyc09g065620	CLH1	–	-1.098	Chlorophyllase 1
Solyc10g006900	POR3	–	-1.450	Protochlorophyllide oxidoreductase
Solyc12g013710	AF243520S1	–	-1.289	Protochlorophyllide oxidoreductase 1

For each GO pathway enriched, the genes belonging to that pathway are shown. log_2_(fold change) (LFC) is reported for genes differentially expressed at T1, or T2, or both. Gene annotation is also reported.

**Figure 6:**
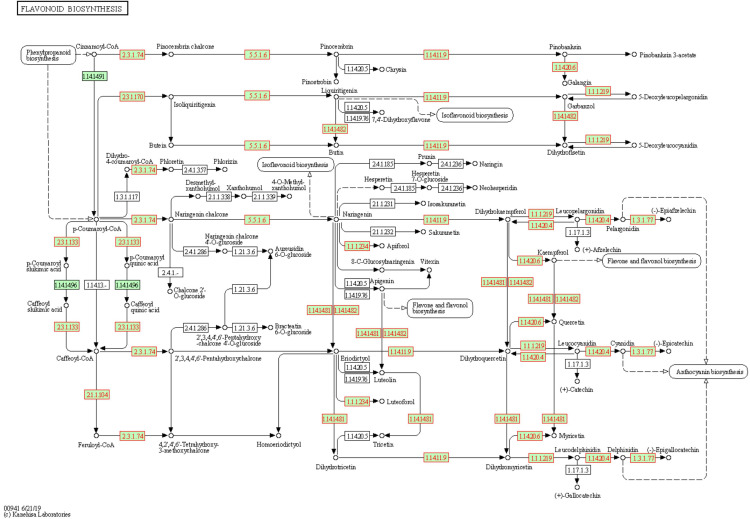
Flavonoids biosynthesis pathway in *Solanum lycopersicum*. Enzymes, chemical compounds, and molecular interactions are represented by boxes, dots, and arrows, respectively. Enzymes synthesized by DEGs are highlighted in red. Enzymes hyperlinked to GENES entries are represented from green boxes.

The KEGG results confirmed those obtained from the GO analysis, with a significant (FDR ≤ 0.05) presence of pathways related to the thiamine, proline, phenylpropanoids, and flavonoids (**Supplementary Table 3**). Again, flavonoids-related pathway resulted significantly enriched at both T1 and T2. Most of the genes annotated in this pathway are differentially expressed (**Figure 6**). Each enzyme is related to one or more genes, as well as the same gene synthesizes one or more enzymes (**Supplementary Table 4**).

### 3.3 Impact on metabolites

Because pathways reated to proline, antioxidant molecules and pigments were significantly enriched according to both GO and KEGG, such compounds were measure. All metabolites were measured at T2, 24 h after the last cold night, in the leaves.

#### 3.3.1 Proline content is influenced by both cold stress and BSE treatment

**Figure 7:**
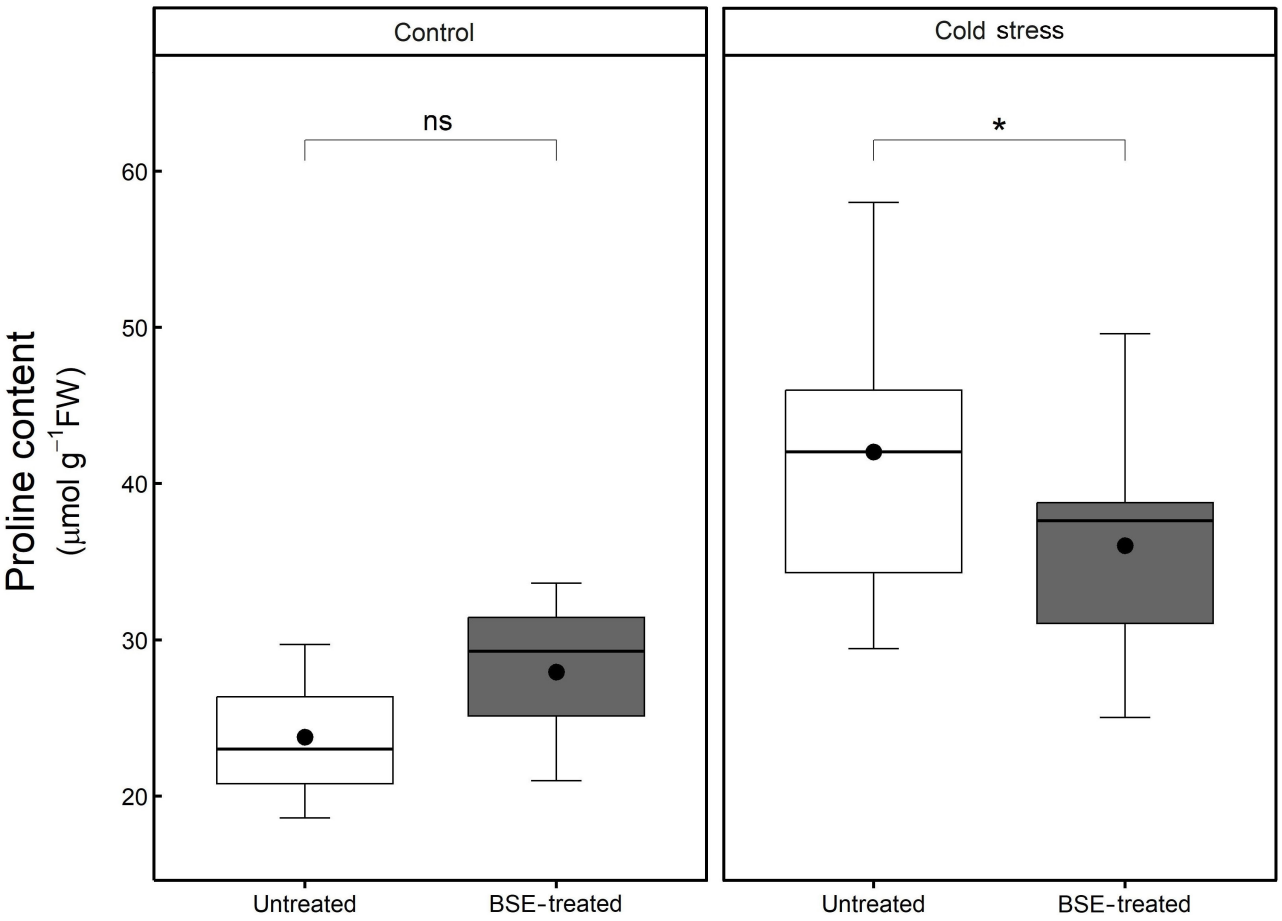
Changes in the content of proline. Proline content in the leaves of tomato plants treated and non-treated, grown in both cold stress and non-stress condition for 3 nights. Box plots show medians, 25^th^ and 75^th^ percentiles, and non-outlier ranges. The dots represent the average values. Significance is based on Wilcoxon’s test: ns, not significant, *p-value < 0.1.

Cold stress increased proline content significantly (p < 0.05) and proportionally more in untreated plants (+76.8%) than in BSE-treated ones (+28.9%) (**Figure 7**). The BSE treatment did not significantly alter proline content (+17.6%) in control plants, but decreased proline content (-14.2%) in cold-stressed ones. These metabolic observations are supporting transcriptomic data (**Figure 7**).

#### 3.3.2 Antioxidant compounds content is influenced by both cold stress and BSE treatment

**Figure 8:**
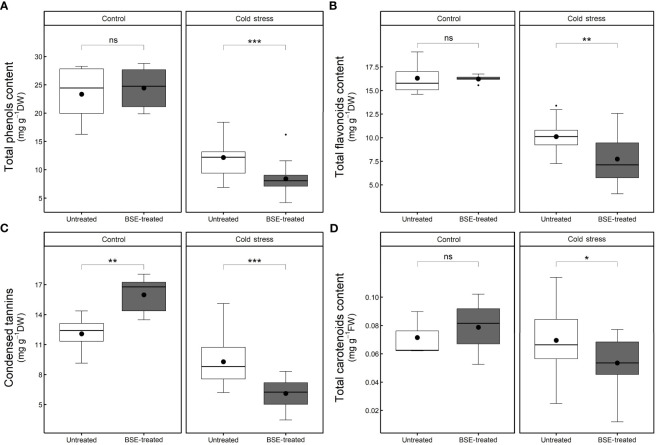
Changes in the content of non-enzymatic antioxidant compounds. Total phenols **(A)**, total flavonoids **(B)**, condensed tannins **(C)**, and total carotenoids **(D)** content in the leaves of tomato plants treated and non-treated, grown in both cold stress and non-stress condition for 3 nights. Box plots show medians, 25^th^ and 75^th^ percentiles, and non-outlier ranges. Small dots are considered outlier observations, big dots represent the average values. Significance is based on Wilcoxon’s test: ns, not significant, *p-value < 0.1, **p-value < 0.05, ***p-value < 0.01.

After 48 h cold treatment, the contents of antioxidant compounds significantly (p < 0.05) decreased both in BSE-treated plants (polyphenols: -65.6%, flavonoids: -52.3%, tannins: -61.7%, and carotenoids: -32.0%) and in untreated ones (polyphenols: -47.8%, flavonoids: -38.0%, tannins: -23.2%, and carotenoids: -2.7%) (**Figure 8**).

In control conditions, the BSE treatment did not modify the contents of polyphenols (+4.7%), flavonoids (-0.5%), and carotenoids (+10.2%), but it significantly (p < 0.05) increased the tannins content (+32.4%). In cold stress conditions, the treatment significantly (p < 0.05) decreased the contents of polyphenols (-30.9%), flavonoids (-23.5%), tannins (-34.1%), and carotenoids (-23.0%) (**Figure 8**).

#### 3.3.3 Chlorophyll content is influenced by cold stress but not from the BSE treatment

**Figure 9:**
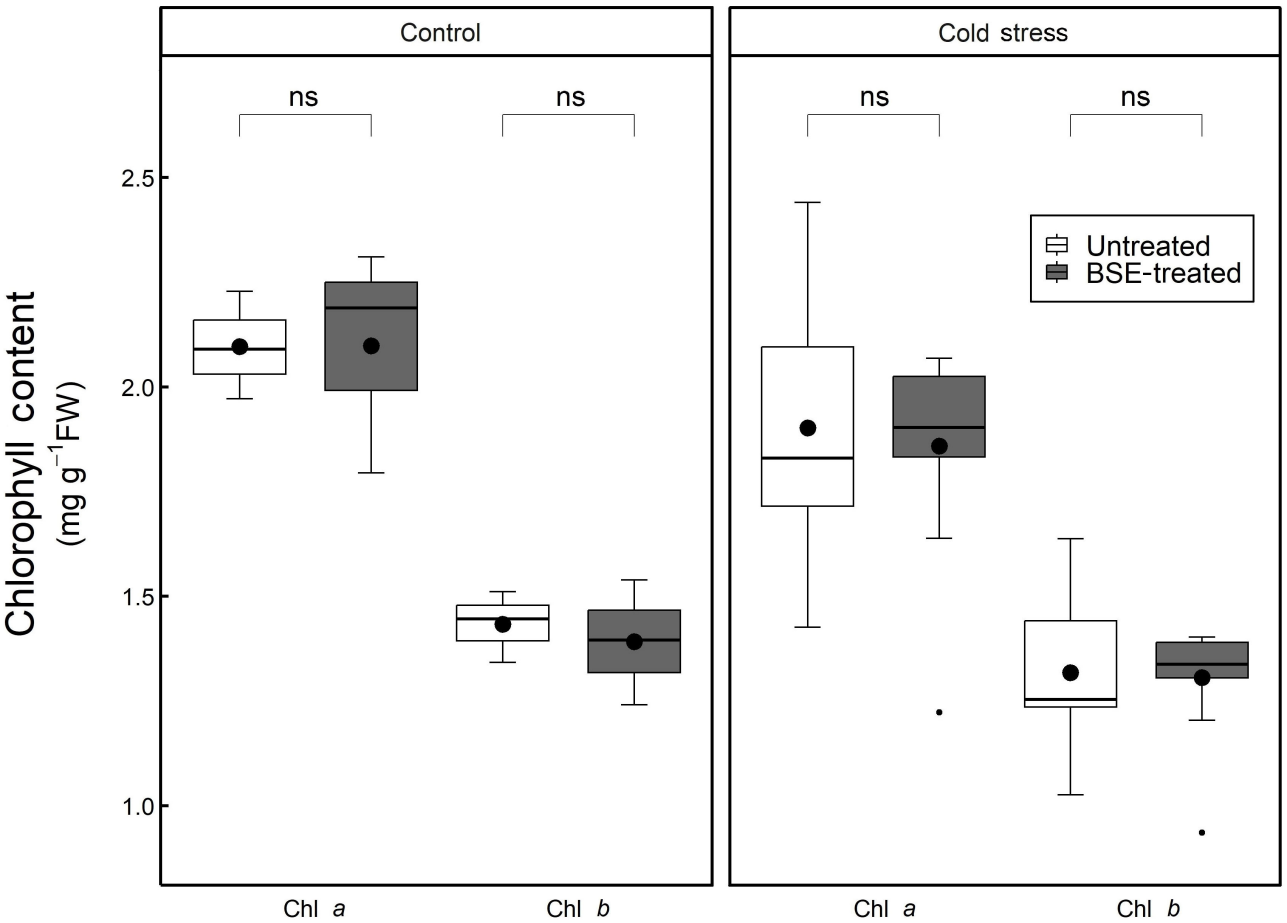
Changes in the content of chlorophyll. Chlorophyll a and b content in the leaves of tomato plants treated and non-treated, grown in both cold stress and non-stress condition for 3 nights. Box plots show medians, 25^th^ and 75^th^ percentiles, and non-outlier ranges. Small dots are considered outlier observations, big dots represent the average values. Significance is based on Wilcoxon’s test: ns, not significant.

The chlorophyll *a* and *b* contents, significantly (p < 0.05) decreased during cold stress both in BSE-treated samples (chl *a*: -11.4%, chl *b*: -6.2%) and in untreated ones (chl *a*: -9.3%, chl *b*: -8.1%) (**Figure 9**). The BSE treatment did not significantly affect the chlorophyll content either in control (chl *a*: +0.1%, chl *b*: -2.9%) or in cold conditions (chl *a*: -2.3%, chl *b*: -0.9%) (**Figure 9**).

## 4 Discussion

Extreme temperatures may lead to important damages to plant growth and development. For instance, cellular membranes are harmed after lipid peroxidation, resulting in electrolyte and amino acid leakage from cells (Hayat et al., 2012). Such biochemical and physiological dysfunctions are mostly caused by the production of ROS. Different actions can mitigate the detrimental effects of low temperatures on crops. Biostimulant products from various origins can improve the plant capacity to tolerate chilling and freezing temperatures (Bulgari et al., 2019; Bhupenchandra et al., 2022). Specifically, algal extracts are known to enhance plant cold tolerance due to their membrane-protective and antioxidative properties (Shukla et al., 2019).

Stomatal conductance and net photosynthesis are commonly used to probe photosynthetic performance and to gauge plant health: they are crucial to determine plant growth and productivity, especially when the plant is undergoing stress conditions. These two parameters are tightly correlated since stomata pores control plant-environment gas exchanges and thus, CO_2_ uptake for photosynthesis (Wong et al., 1979; Damour et al., 2010). Under control and cold conditions, the BSE treatment increased both stomatal conductance and net photosynthesis (**Figures 1A, B**), as previously reported (Baghdadi et al., 2022). This increase in physiological activity may explain the improved yield shown by treated tomato plants, even under cold stress. Again, the yield of cold-stressed BSE-treated plants was comparable to that of untreated plants which did not face any stress (**Figure 2**). At each of the three time points, physiological (stomatal conductance and net photosynthesis), and yield parameters (fruit number, diameter, fresh and dry weight) of the BSE-treated plants showed an improvement with respect to control plants.

One PCA (**Figure 3**) confirmed this assumption considering as a worst-case scenario untreated-cold-stressed plants (clustering at the extreme left) and as a best-case scenario BSE-treated-unstressed plants (clustering at the extreme right). The analysis exhibits a gradient that runs from the worst-case scenario to the best-case scenario: intermediate situations (BSE-treated stressed plants and untreated-unstressed plants) are overlapping.

**Figure 10:**
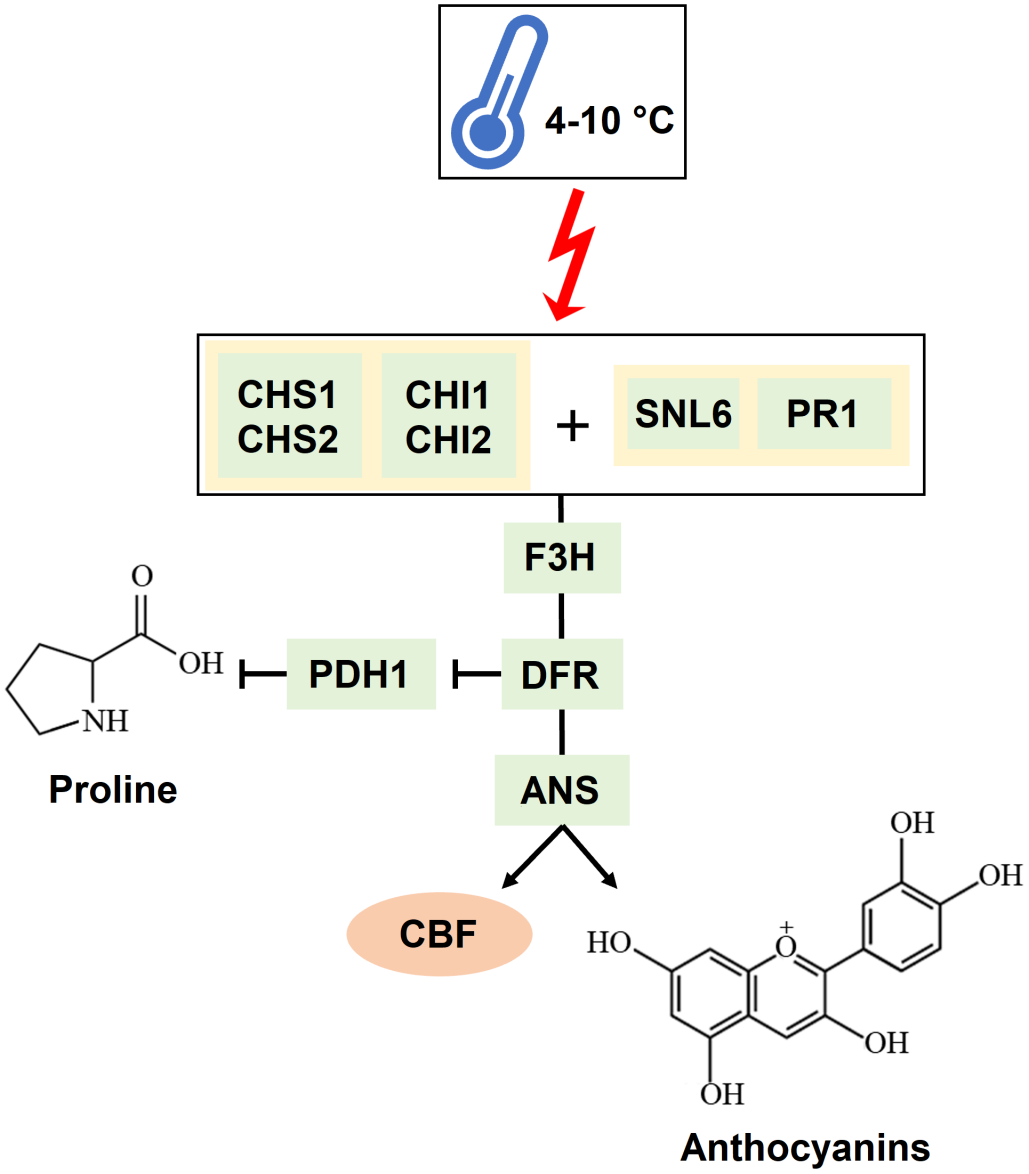
Mechanism of interaction of the genes identified as significantly involved in the cold response. A temperature ranging between 4 and 10°C triggers *CHALCONE SYNTHASES* (*CHSs*) and *CHALCONE-FLAVONONE ISOMERASES* (*CHIs*) together with *PATHOGENESIS-RELATED PROTEIN 1a* (*PR1*) and *CINNAMOYL-COA REDUCTASE-LIKE 6* (*SNL6*). These genes trigger the activation of *FLAVANONE 3-HYDROXYLASE* (*F3H*), *DIHYDROFLAVONOL 4-REDUCTASE* (*DFR*), and *ANTHOCYANIDIN SYNTHASE* (*ANS*). *DFR* also inhibits *PROLINE DEHYDROGENASE* (*PDH1*), which degrades proline. The result of this cascade is the production of anthocyanins and the activation of the *CBF* transcription factor.

A global transcriptomic analysis was conducted to characterize the action modes of BSE in mitigating cold stress. A total of 1.238 DEGs were identified at two time points (T1 = 24 h and T2 = 48 h) during the stress-recovery phase. Ten genes were downregulated at T1 but upregulated at T2, while 20 genes followed the opposite pattern (**Table 1**). Falling into the first category, *CHALCONE SYNTHASE 1;2* (*CHS1;2*), *CHALCONE FLAVONE ISOMERASE 1*;*2* (*CHI1*;*2*), *FLAVONE 3-HYDROXYLASE* (*F3H*), *DIHYDROFLAVONOL 4-REDUCTASE* (*DFR*) and *ANTHOCYANIDIN SYNTHASE* (*ANS*) are involved in the flavonoid biosynthetic pathway. These genes are also activating the *C-REPEAT BINDING FACTORS* (*CBFs*) pathway, leading to anthocyanin production (He et al., 2022) (**Figure 10**). More generally, genes in the flavonoid pathway interact with some pathogen-related (PR) genes in response to various stress conditions (Dai et al., 2022). In particular, *CHS*, *CHI*, and *F3H* are depicted as central regulators during the cold response in tomato (Han et al., 2020). The modulation of defense signaling pathways mediated by salicylic acid (SA), jasmonic acid, or ethylene is well documented after treatment with BSE in plants (Ali et al., 2021). Triggering these signaling pathways increases the expression levels of *PR* genes and genes encoding defense enzymes involved in the synthesis of polyphenolic compounds with anti-pathogenic properties (Vera et al., 2011). Nonetheless, SA plays a role in plant protection against abiotic stress, including cold (Liu et al., 2022). That hormone regulates the activity of antioxidative enzymes (Wani et al., 2017). Exogenous SA application can activate the alternative oxidase in sweet pepper exposed to cold (Fung et al., 2004), and improve chilling tolerance in cucumber through the cold-signaling pathway activation (Fu et al., 2021). Moreover, SA triggers the accumulation of soluble sugars and proline during cold or heat temperature stress, promoting tolerance through antioxidant and osmotic regulation (Soliman et al., 2018; Jahan et al., 2019). The DE genes *PR1* and *SNL6* thus, can be considered not only involved in biotic stress defence, but also in abiotic stress response (Xu et al., 2021; Akbudak et al., 2020; Yin et al., 2023) (**Figure 10**).

Indeed, GO analysis highlighted proline, antioxidants, and pigment pathways as highly significantly responsive to the BSE treatment.

Proline is an amino acid that accumulates in plant tissue during stress (Trovato et al., 2008). Leaf proline content is following the tendency illustrated in the PCA: control plants (BSE-treated and untreated) have a lower proline content than cold-stressed BSE-treated plants, which accumulated less proline than cold-stressed untreated plants (**Figure 7**). We can suppose that stressed-BSE-treated plants are less affected by the low-temperature stress compared to stressed untreated plants, showing an average situation (but still statistically significant) between them and the unstressed ones. Proline plays a plethora of roles in stress response. Apart from being an antioxidant molecule, it acts as an osmolyte maintaining membranes and protein structures (Ashraf and Foolad, 2007). The present BSE-based biostimulant contains proline (Lakshmi and Meenakshi, 2022), and low exogenous proline increases plant tolerance toward various stresses (Ashraf and Foolad, 2007; Hayat et al., 2012). So the greater proline content could be a result of an exogenous application. However, in the enriched proline pathway, according to GO, a proline dehydrogenase (PDH1) is present. PDH1 has been reported to act with a mitochondrial protein, DFR1, in response to cold stress. Briefly, DFR interferes with PDH1 (and PDH2) to prevent proline degradation and increment its accumulation (Ren et al., 2018) (**Figure 10**). A lower amount of proline was measured in BSE-treated plants in respect to untreated ones, under cold stress. This can be due to the antioxidant power of the amino acid: in fact, during cold stress the proline content increases and then decreases (Azami et al., 2021). Or it can be caused by the modulation of the PDH1 gene. PDH1 was downregulated by the treatment under cold stress at T1, while its opposer, DFR, was downregulated at T1 and upregulated at T1 (**Tables 1**, **2**). A quantification of the proline amount over the time of a stress would be needed to better understand the behavior of this protein in response to both the stress and the treatment.

There are no available data about the pattern of accumulation of antioxidants over time during cold exposure and recovery in tomato. At an early stage, the plant produces enzymatic and non-enzymatic antioxidants (e.g., flavonoids and polyphenols) to scavenge ROS burst (Rezaie et al., 2020). Free radicals generated during cold stress overstep the plant antioxidant capacity, and this leads to an oxidative stress (Hayat et al., 2012). The quantity of antioxidants then decreases as the stress progresses because of the reaction with the ROS: antioxidants prevent the oxidation of biomolecules by supplying the electrons needed (Azami et al., 2021). In the case of phenols, the resulting oxidized molecules, the benzoquinones, are unstable and need to be worked off (Barmaverain et al., 2022): indeed, the Folin-Ciocalteu method, quantifying the total phenols, can be considered as a measure of the antioxidant capacity of the plant (Platzer et al., 2021; Rumpf et al., 2023). Antioxidants were quantified only at T2, and they resulted significantly lower in the cold-stressed plants than in the non-stressed ones. Moreover, they resulted significantly lower in the cold-stressed BSE-treated plants, than in the cold-stressed untreated ones (**Figure 8**). Because the performances of cold-stressed BSE-treated plants were better than those of cold-stressed untreated ones, from the physiological point of view, this lower quantity of antioxidants in the treated plants can be the result of a higher antioxidant capacity of the BSE-treated plants. Anyway, a quantification of the antioxidant compounds over the time of a cold wave would be needed to understand tomato response to this stress. Anthocyanins are a class of flavonoids which are a class of polyphenols thus, they were included in the total phenols and flavonoids measurement. Indeed, flavonoids metabolism was found to be significantly regulated by the BSE treatment, and the genes in this GO class (CHS1, CHS2, F3H, ANS) are part of the anthocyanins biosynthetic pathway (**Figure 10**). Genes involved in the antioxidants biosynthetic pathways are very often inverting their expression tendency from T1 to T2, this could be due to an adjustment of antioxidant amounts after the stress. A lot of transcription factors are implied in this process (He et al., 2022) but were not highlighted in the analysis. Anyway, it was possible to identify a consistent amount of genes regulated by the BSE application.

Although the GO analysis pointed to an over-representation of the chlorophyll metabolic process, no significant difference in the chlorophyll content was measured following BSE application or cold exposure. Still, genes encoding enzymes degrading the chlorophyll were down-regulated after BSE treatment at T2 (**Table 2**). The free radicals generated can degrade chlorophyll (Sharma et al., 2020), but antioxidant molecules can also play an opposite role in this process, protecting the pigment from that (Leòn-Chan et al., 2017). This could be the reason for observing no alteration in chlorophyll content after cold stress. Anyway, it is hard to explain the role of downregulated chlorophyllases in our analysis. Generally, BSE-treated plants seem to have a higher content of both chlorophyll a and b, but these differences are not significant.

In conclusion, we mimicked a late cold snap, with temperature dropping at night and rising during the day. Three BSE applications until BBCH65 efficiently protected tomato plants, by increasing yield and reducing fruit cracking. The BSE treatment seems not to directly target the CBF/ICE regulatory pathway but rather the antioxidative molecule production to protect plants against cold stress. These findings on BSE treatment could have important implications for tomato cultivation, but also in a more general context, for crop productivity and protection.

## Data availability statement

The datasets presented in this study can be found in online repositories. The names of the repository/repositories and accession number(s) can be found below: https://www.ebi.ac.uk/ena, PRJEB62653.

## Author contributions

FMan, FMag, AM, PS: conceptualization. PS, FMan, SN, AM: supervision. FMan, PS, SC, AM, AB, MCDL, MB, GB, WZ-L, CC, EP, CH, AliB: methodology. MB, MCDL and CC: writing the original draft. MB: data analysis and graphical representation. FMan, CC, SN, CH, PS, and SN: writing, reviewing, and editing. All authors contributed to the article and approved the submitted version.

## Supplementary material

Supplementary Figure 1 Volcano plots of the DEGs at the two time points. Volcano plot at T1 **(A)** and T2 **(B)**. Volcano plots show the DEGs in terms of LFC and p-value. Dotted lines represent the chosen thresholds: |LFC| > 1 and p-value < 0.05. Green and orange dots refer to non-significant genes. Blue and red dots refer to downregulated and upregulated genes above the p-value thresholds, while purple dots refer to DEGs which are above the LFC threshold as well.

Supplementary Figure 2 Samples overview. Euclidian distance heatmap **(A)** showing samples clustering based on their normalized expression patterns. Top colored bars show treatmen and sampling times variables. PCA at T1 **(B)** and T2 **(C)** show samples colored according with the treatment (green = untreated, orange = BSE-treated).
