# Supplementary figures and images for: Transcriptomic and physiological approaches to decipher cold stress mitigation exerted by brown-seaweed extract application in tomato
Source: Front Plant Sci. 2023 Sep 11;14:1232421. doi: 10.3389/fpls.2023.1232421 (PMC10520554; doi:10.3389/fpls.2023.1232421)

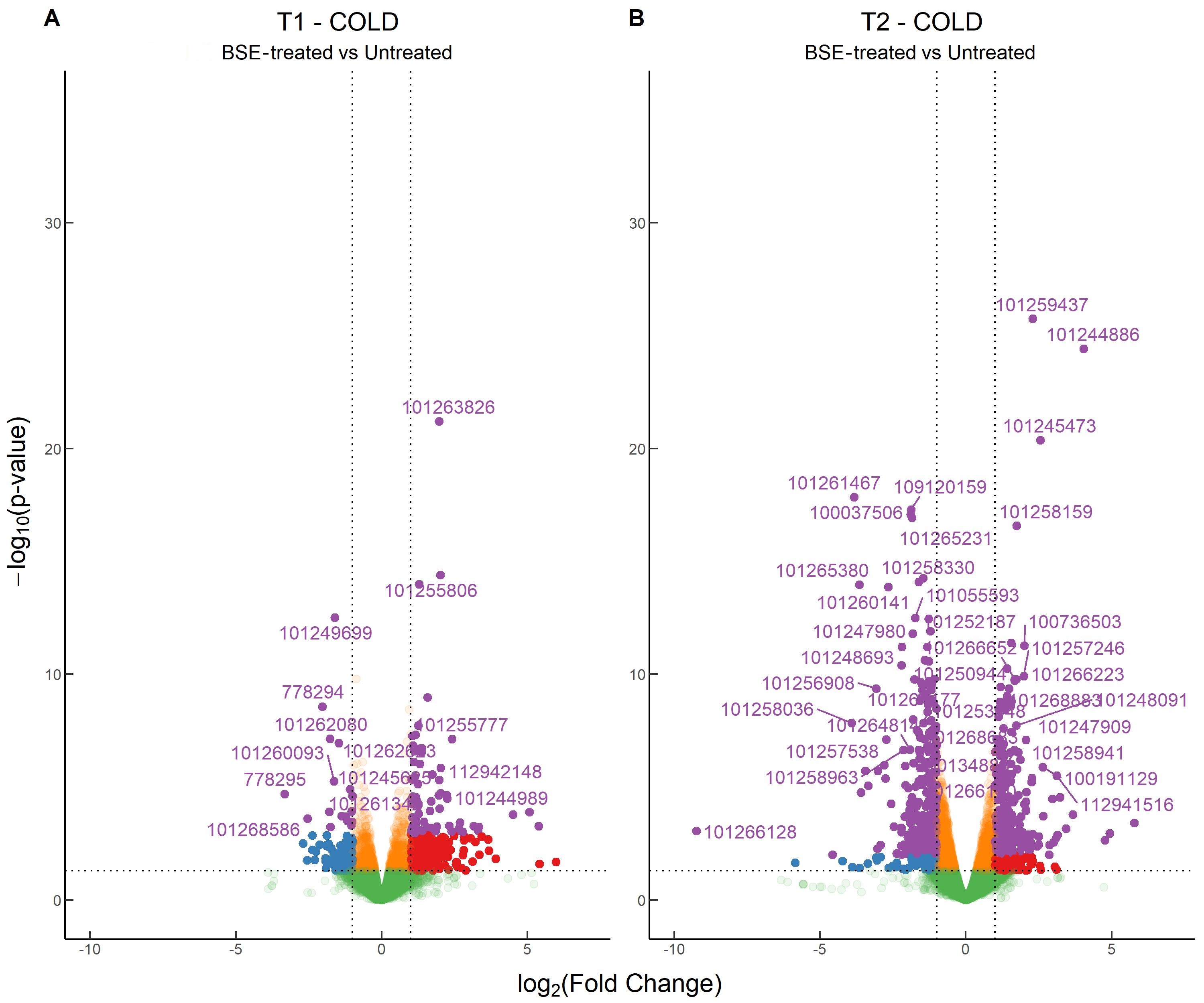

Supplement: Supplementary Figure 1 — Volcano plots of the DEGs at the two time points. Volcano plot at T1 (A) and T2 (B). Volcano plots show the DEGs in terms of LFC and p-value. Dotted lines represent the chosen thresholds: |LFC| > 1 and p-value < 0.05. Green and orange dots refer to non-significant genes. Blue and red dots refer to downregulated and upregulated genes above the p-value thresholds, while purple dots refer to DEGs which are above the LFC threshold as well. [file Image_1.tiff]

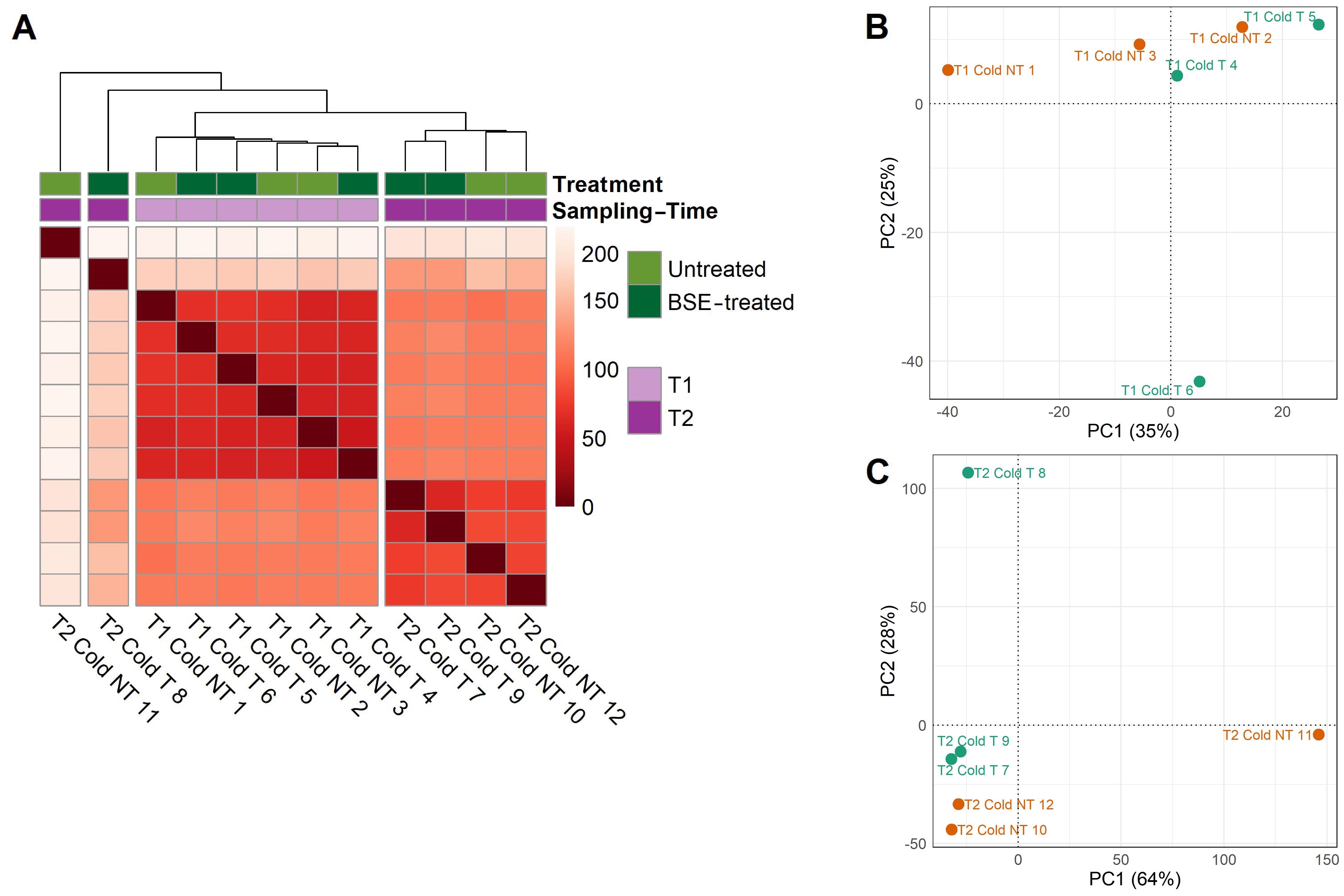

Supplement: Supplementary Figure 2 — Samples overview. Euclidian distance heatmap (A) showing samples clustering based on their normalized expression patterns. Top colored bars show treatmen and sampling times variables. PCA at T1 (B) and T2 (C) show samples colored according with the treatment (green = untreated, orange = BSE-treated). [file Image_2.tiff]
